# Case Report: Mature congenital teratoma masquerading as orbital cellulitis in a 12-day-old infant

**DOI:** 10.3389/fopht.2025.1689096

**Published:** 2025-10-15

**Authors:** Alyssa C. Huelsbeck, Colin P. Froines, Suzanne W. van Landingham

**Affiliations:** 1 School of Medicine and Public Health, University of Wisconsin, Madison, WI, United States; 2 Department of Ophthalmology and Visual Sciences, University of Wisconsin, Madison, WI, United States

**Keywords:** congenital teratoma, orbital cellulitis, neonatal orbital mass, case report, proptosis, orbitotomy

## Abstract

**Background:**

Congenital orbital teratoma is a rare neoplasm that typically presents as progressive, unilateral proptosis in an otherwise healthy newborn. Management includes prompt surgical excision, with guarded visual prognosis but excellent survival.

**Case presentation:**

A 12-day-old healthy infant presented with progressive left eye swelling and proptosis. She was initially diagnosed with orbital cellulitis and treated with IV antibiotics. Magnetic resonance imaging (MRI) showed a 1.5x1.9x2.1 cm left intraconal mass with 9mm of proptosis and significant mass effect. The patient underwent left lateral orbitotomy for biopsy and excision of the mass. Histopathologic examination showed neutrophilic inflammation and granulation tissue with foci of gastrointestinal epithelium, cartilage, squamous epithelium, and ganglion cells, consistent with mature congenital teratoma. The postoperative course was uncomplicated and there is no sign of recurrence at 21 months of age.

**Conclusion:**

Orbital teratoma should be suspected in a rapidly growing orbital mass in a newborn. Imaging showing characteristic findings should lead to prompt excisional biopsy. Tumor markers can be used to monitor for recurrence, which is rare.

## Introduction

The differential diagnosis for rapidly worsening proptosis in a newborn includes orbital cellulitis, lymphatic or vascular malformations, teratomas, dermoid cyst, rhabdomyosarcoma, and metastatic neuroblastoma (1). In a retrospective analysis, Sindhu et al. (2) showed that infectious orbital cellulitis was the most common cause of proptosis in children under 15 years of age, typically presenting with pain, eyelid erythema, chemosis, and proptosis with or without fever. Distinguishing the etiology of orbital processes can be difficult and typically requires imaging.

Teratomas are rare congenital neoplasms that contain tissue derived from all three germ layers: ectoderm, mesoderm, and endoderm (3). Most teratomas occur in the gonads or coccyx, with orbital teratoma representing 0.8% of cases. Roughly 100 cases have been presented in the literature (4). They are more common in girls and in left orbits, and typically present as progressive, unilateral proptosis in an otherwise healthy newborn (3, 4). Teratomas can be classified as mature or immature, with immature teratomas containing immature neuroepithelial tissue (5). Immature teratomas are considered malignant and may invade adjacent tissues (6). Mature teratomas are typically benign though they can occasionally show malignant degeneration (7).

We present here a case of mature congenital teratoma masquerading as orbital cellulitis in a 12-day-old infant. Collection and evaluation of protected patient health information was in compliance with the Health Insurance Portability and Accountability Act of 1996 and adhered to the ethical principles outlined in the Declaration of Helsinki as amended in 2013.

## Case description

A 12-day-old female presented with one day of rapidly worsening left eye swelling and proptosis. The patient was born full-term with an uncomplicated pregnancy and vaginal delivery. She was Group B strep negative, maternal history was negative for history of gonorrhea or chlamydia, and she received prophylactic erythromycin ointment at birth. Antenatal anatomy untrasound at 20 weeks showed no ocular or facial abnormalities. Her family history was negative for known consanguinity, congenital or ocular tumors, or teratogenic exposures. There was no suspicion for ocular abnormality at birth.

**Figure 1:**
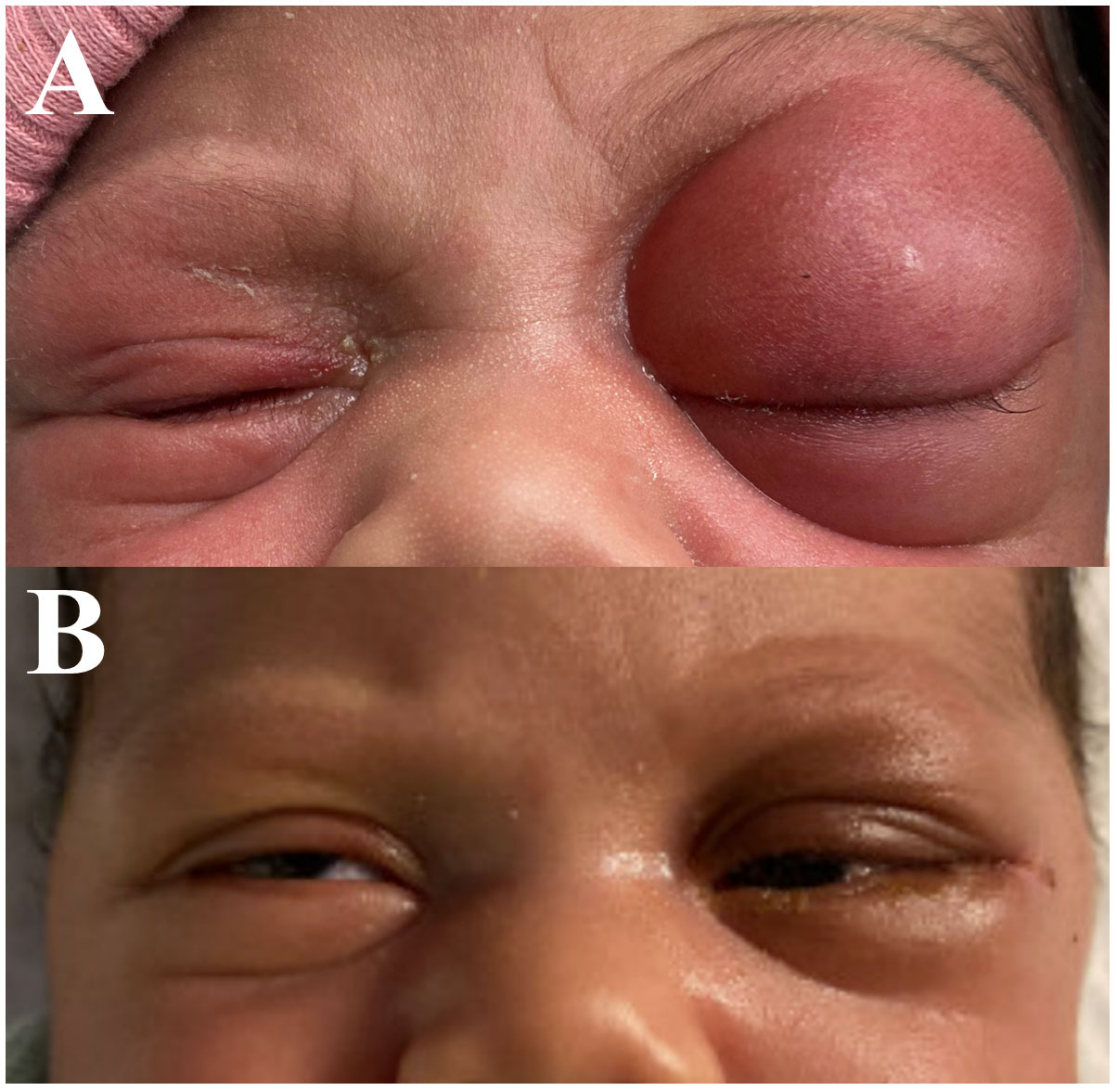
External photograph of 12-day-old female demonstrating left eye swelling and proptosis shown preoperatively **(A)** and 5 days postoperatively following lateral orbitotomy **(B)**.

At the time of her presentation to Urgent Care, examination of the left eye was notable for proptosis, chemosis, severe edema, and erythema of the upper and lower eyelid (**Figure 1A**). No purulent discharge was expressed with palpation of the lacrimal sac or canaliculi. Pupils were equal, round, and reactive to light bilaterally with no relative afferent pupillary defect (RAPD). Intraocular pressure was 32 in the right eye and 30 in the left eye. Examination of the right eye was otherwise within normal limits.

Laboratory evaluation was performed with basic metabolic panel showing slightly elevated calcium (11.6 mg/dL) and complete blood count with mild anemia (hemoglobin 13.8 g/dL), monocytosis (4,280/uL), and thrombocytosis (584 K/uL). C-reactive protein, procalcitonin, neutrophils, and lymphocytes were within normal limits. The patient was diagnosed with orbital cellulitis and admitted for intravenous antibiotics (ceftazidime and vancomycin).

**Figure 2:**
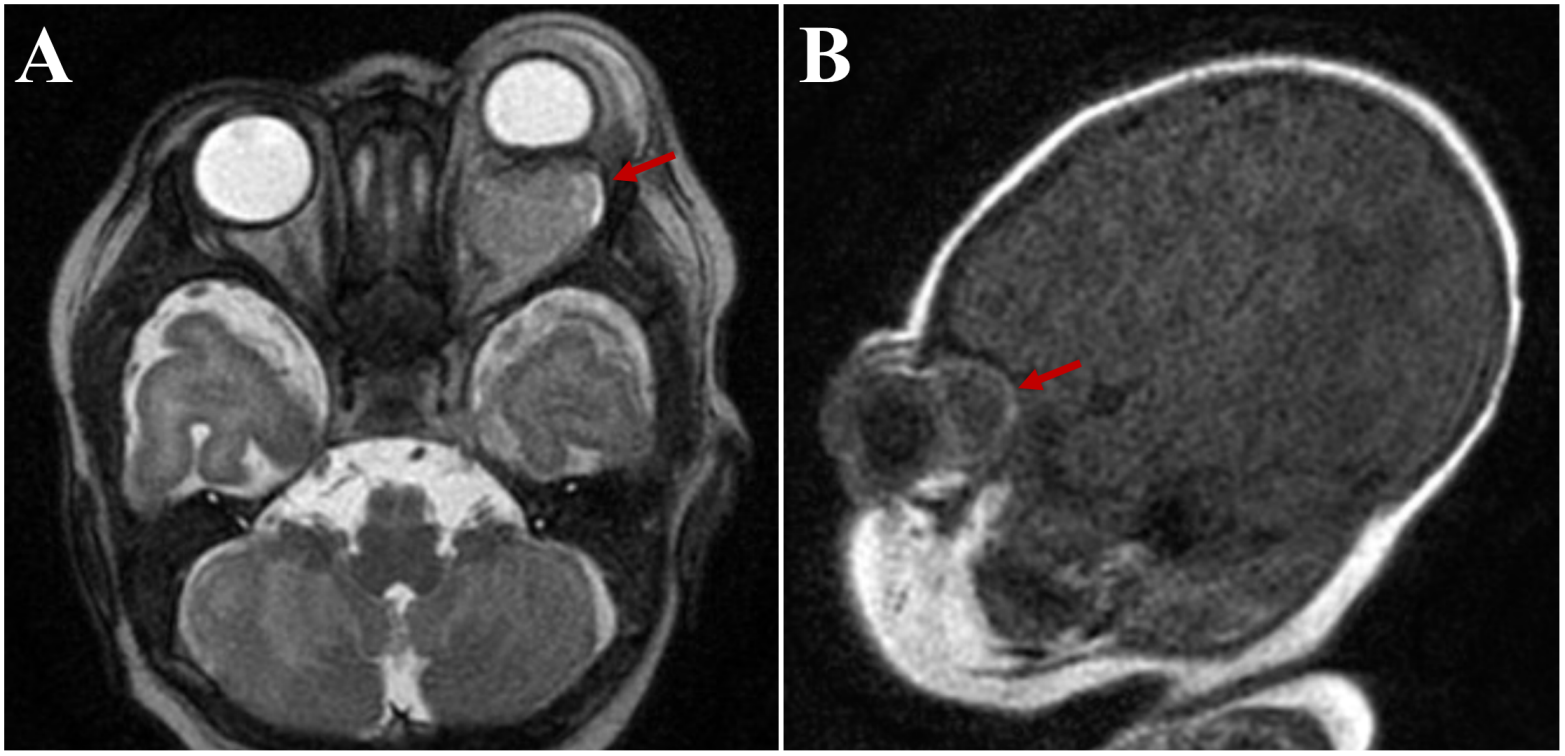
Axial **(A)** and sagittal **(B)** magnetic resonance images, demonstrating a 1.5x1.9x2.1 cm left intraconal mass initially read as “abscess” (arrows).

Magnetic resonance imaging (MRI) was obtained to rule out abscess. It showed a 1.5x1.9x2.1 cm left intraconal mass with significant mass effect displacing the optic nerve and 9 mm of proptosis. Diffuse pre-septal soft tissue edema was also noted (**Figure 2**). This was initially interpreted by the reading radiologist as an abscess, however consultation with the orbital surgeon raised concerns for cystic mass such as lymphatic malformation or teratoma.

**Figure 3:**
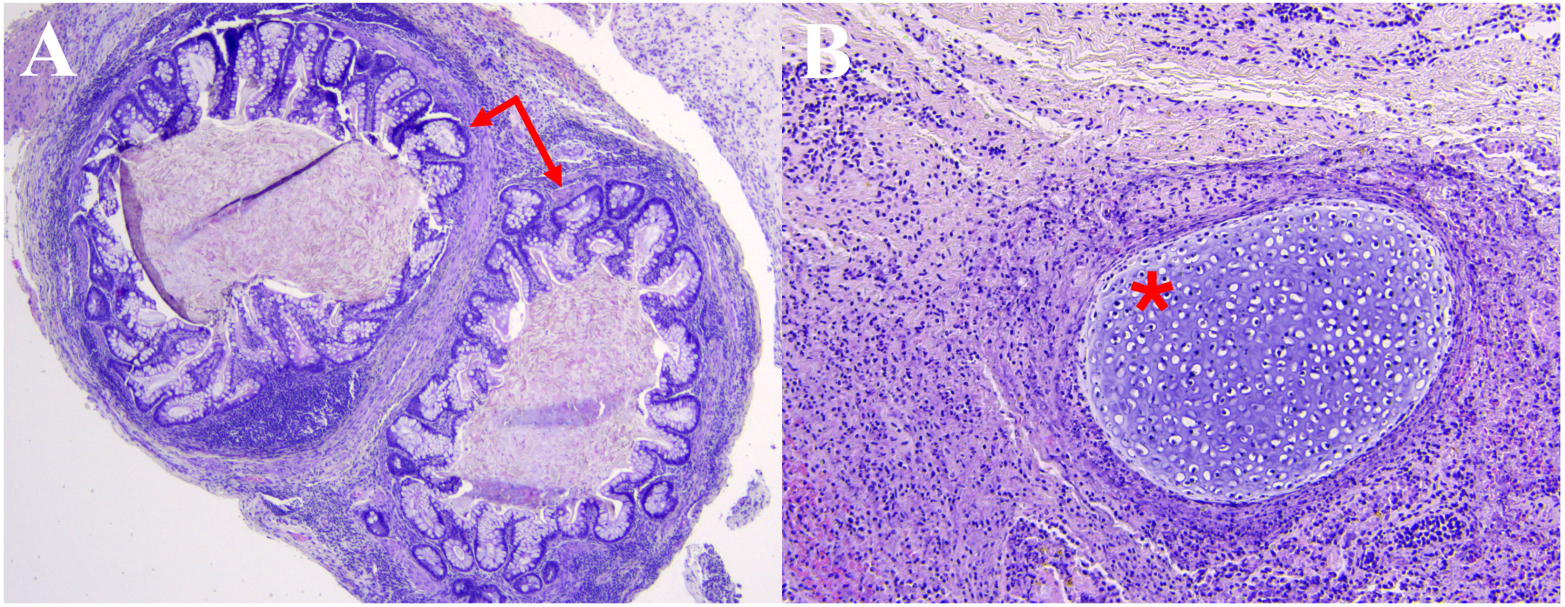
Hematoxylin and eosin histopathology slides demonstrating foci of **(A)** gastrointestinal epithelium (arrows) and **(B)** cartilage (*) among neutrophilic inflammation and associated granulation tissue.

Within 24-hours of presentation the patient underwent left lateral orbitotomy with operative goal to perform globe-sparing mass debulking and biopsy for tissue-based diagnosis. Intraoperatively, the mass was accessed via broad subperiosteal dissection along the lateral orbital wall. Careful blunt dissection revealed the mass to be cystic, white, and ballotable. Further careful dissection was employed to avoid inadvertently traumatizing the optic nerve. Once exposure was optimized, a piece of the mass was excised and copious milky fluid egressed from the mass which was cultured. Additional blunt dissection was performed to dissect the mass which was found to have multiple loculations and measured 25mm x 22mm x 5mm which was sent en bloc for histopathological analysis. Following the excision the orbit was irrigated copiously with a drain placed in the lateral orbit. Histopathological evaluation showed neutrophilic inflammation (CD45 and myeloperoxidase positive) and granulation tissue with foci of gastrointestinal epithelium (**Figure 3A**), cartilage (**Figure 3B**), squamous epithelium, and ganglion cells, consistent with mature congenital teratoma. Cultures and 16s rRNA sequencing were negative.

The early postoperative course was uncomplicated. Orbital edema improved dramatically with three days of intravenous corticosteroids and surgical drain was removed on post-operative day 4 (**Figure 1B**). The patient’s case was discussed by a multidisciplinary pediatric tumor board and is monitored by the pediatric oncology service with serial tumor marker levels (alpha fetoprotein (AFP), human chorionic gonadotrophin (HCG), and lactate dehydrogenase (LDH) immediately, then at 3, 6, 12, and 18 months of age) and surveillance MRIs at 3, 6, and 12 months of age with no sign of disease recurrence at 22 months of age.

She required strabismus surgery for esotropia at 12 months of age, and her eye exam is notable for optic nerve pallor and a trace APD on the left side consistent with compressive optic neuropathy. Her fixation is central, steady and maintained in the right eye, unsteady, steady, and unmaintained in the left eye. Visual evoked potential testing demonstrates greater visual function in the right eye compared to the left, with 8 and 6.5 cycles per degree respectively. Cycloplegic refraction shows symmetric hyperopia of +4.50 in both eyes. Patient is followed for amblyopia and undergoing patch therapy. Visual prognosis is guarded.

## Discussion

We describe herein a rare case of mature congenital teratoma in a 12-day-old infant masquerading as orbital cellulitis. Demonstration of intraconal mass on MRI prompted orbitotomy, which led to histopathological diagnosis. Prompt diagnosis and treatment led to successful surgical excision sparing the globe, though visual prognosis is guarded.

Imaging can be used to characterize orbital masses: CT or MRI imaging of congenital teratoma shows well-circumscribed, multiloculated, heterogeneous cystic masses (1). Histopathology is needed for definitive diagnosis of teratoma. Management involves early surgical excision with orbital exenteration or globe-sparing surgeries (4).

The neutrophil-to-lymphocyte ratio (NLR) (8) is emerging as a biomarker that may be useful in distinguishing infectious states from masqueraders. The ratio compares the innate immune system response (represented by neutrophil count), which is typically elevated in acute infection, with the adaptive immune response (represented by lymphocyte count). While cut-off levels are still being developed, an NLR over 5 has been associated with infection and metastatic cancer. In the orbit, Wladis et al. (9) have proposed using the NLR as a biomarker to distinguish between infectious and inflammatory orbital disease, with a high NLR suggesting orbital infection. The NLR in this patient at presentation was 2.2, which would be consistent with a non-infectious etiology. Consideration of NLR at the time of presentation could help to distinguish teratoma cases from cellulitis cases, particularly when used in conjunction with other findings due to the lack of well-established cut-offs.

Pediatric patients with a small-to-moderate sized subperiosteal abscess may be managed medically with intravenous antibiotics, but surgical drainage is indicated when the abscess is large, intraconal, and causing proptosis (10). Management of orbital teratoma requires prompt surgery aiming for complete excision. Management with orbital exenteration is sometimes reported (11), particularly in cases with a large tumor causing severe consequences such as exposure-related perforation (12), but globe-sparing surgeries are now typically possible (1, 4). Size at presentation, early recognition and surgical intervention aided in the success of globe-sparring surgery in this case. Tsoutsanis et al. reported a case of an impressively large teratoma excised with a globe-sparing approach: the patient kept her globe, though not her vision, for the entire 18-year follow-up period (1). Ming et al. describe a case of a smaller teratoma managed with a globe-sparing approach in which the patient is visually attentive at 6 months. Given that management differs significantly from other diagnoses and prompt treatment is crucial for favorable visual outcomes, early recognition of orbital teratoma is necessary (4).

Surgical excision is curative for most patients with mature congenital teratoma. Malignant changes are seen within <3% of mature congenital teratomas (7), and approximately 4.2% of mature teratomas recur (13). Both of these values are calculated from series of ovarian teratomas, as large case series of the rarer orbital teratoma are not available. It is unclear if orbital teratomas have the same rates of malignancy and recurrence, though it is plausible that they do, given their histopathologic similarities.

Our patient underwent surveillance imaging and laboratory testing of tumor markers to monitor for recurrence. The role of tumor markers is somewhat unclear (14). Elevated AFP, HCG, and LDH are associated with germ cell tumors like teratomas. AFP, in particular, is quite high at birth and then drops precipitously in the neonatal period (15). Our patient’s initial AFP value was 4,143.5 ng/mL, which is within the normal range for a 2 week old infant. Her lab draws at 3, 6, and 12 months of age decreased to 217.4, 61.8, and 4.3, respectively, which are all within normal range for age. A spike in AFP would suggest teratoma recurrence. Given the speed at which a teratoma can grow, the patient’s family were also issued return precautions: progressive periorbital edema, erythema, or proptosis should trigger immediate evaluation.

In conclusion, orbital teratoma should be considered in a rapidly growing orbital mass in a newborn. Imaging showing characteristic findings should lead to prompt excisional biopsy. Visual prognosis is poor as the expanding mass may cause a compressive optic neuropathy, but survival prognosis is excellent given the rarity of malignant transformation. Limitations of this case include description of a single case, limited follow-up, and absence of genetic testing.

## Data Availability

The datasets presented in this article are not readily available because this is a case report. Identifiable information may not be shared. Requests to access the datasets should be directed to SL, svanlandingh@wisc.edu.
